# A decision tree analysis to predict massive pulmonary hemorrhage in extremely low birth weight infants: a nationwide large cohort database

**DOI:** 10.3389/fped.2025.1529712

**Published:** 2025-03-21

**Authors:** Kyu Hee Park, Eun Yeob Kim, Hye Won Cho, Jong Ki Jung, Yu Seon Kim, Byung Min Choi

**Affiliations:** ^1^ Department of Pediatrics, Korea University Ansan Hospital, Ansan-si, Republic of Korea; ^2^ Medical Science Research Center, Korea University Ansan Hospital, Ansan-si, Republic of Korea; ^3^ Department of Pediatrics, Woori Children’s Hospital, Seoul, Republic of Korea; ^4^ Department of Pediatrics, College of Medicine, Korea University, Seoul, Republic of Korea

**Keywords:** pulmonary hemorrhage, mortality, risk factor, preterm infant, decision tree analysis

## Abstract

**Objective:**

To develop a decision tree model using clinical risk factors to predict massive pulmonary hemorrhage (MPH) and MPH-related mortality in extremely low birth weight infants (ELBWIs).

**Method:**

We retrospectively analyzed data from a national multicenter prospective web-based registry using machine learning algorithms with the C5.0 decision tree model to develop a clinical prediction rule for MPH and MPH-related mortality in ELBWIs admitted to participating neonatal intensive care units (NICUs) from January 2013 to December 2020. This C5.0 model was developed through data preprocessing, attribute selection based on splitting criteria, and pruning techniques to minimize overfitting.

**Results:**

A total of 5,752 infants were included. Of them, MPH occurred in 664 (11.5%) infants. Among infants with MPH, 136 (20.5%) infants died due to MPH. The decision tree model for MPH identified “gestational age (GA) ≤ 25^+2^” as the first discriminator, followed by “APGAR score at 5 min ≤ 7” and “multiple gestation”. The decision tree model for MPH-related mortality identified “GA ≤ 25^+2^” as the first discriminator, followed by “APGAR score at 5 min ≤2”. The predictive accuracy of the C5.0 MPH model achieved an area under the ROC curve (AUC) of 88.2% on the training set and 89.0% on the test set, while the MPH-related mortality model attained an AUC of 97.7% on the training set and an AUC of 97.4% on the test set.

**Conclusions:**

We developed a C5.0 decision tree model using clinical risk factors to predict MPH and MPH-related mortality in ELBWIs, enabling early identification of high-risk infants and facilitating timely interventions to improve neonatal outcomes. This decision-based risk stratification tool requires additional verification using larger multicenter cohorts to evaluate its practical applicability and clinical effectiveness before routine clinical implementation in NICUs.

## 1 Introduction

Massive pulmonary hemorrhage (MPH) in neonates is a critical and life-threatening condition characterized by a rapid onset of significant pulmonary bleeding, which can lead to severe cardiorespiratory instability. In extremely preterm infants, MPH remains a life-threatening emergency with high risks of neonatal death and increased risks of long-term morbidities despite various management approaches (1, 2). The incidence of MPH varies by birth weight and diagnostic criteria, ranging from 5% to 8% in very low birth weight infants (VLBWIs), with a recent report showing an incidence of 6.3% in Korea (2, 3). Therefore, identifying risk factors for MPH and predicting associated complications are crucial for enhancing survival rates and quality of life of these infants.

Previous studies identifying risk factors for MPH have mainly utilized statistical methods such as logistic regression (4–7). Recently, our researchers have analyzed a large, nationwide cohort database to identify perinatal risk factors for MPH and MPH-related mortality in very low birth weight infants (VLBWIs) and proposed proactive management strategies to reduce MPH incidence and associated mortality (3).

Although traditional statistical methods (both univariate and multivariate analyses) can identify significant variables, they are limited in determining the relative importance or priority of these variables. In contrast, decision tree analysis as part of a clinical decision support system offers a clear advantage by confirming the priority of significant variables based on their contribution to the outcome, providing a more intuitive and structured understanding of the data.

Similarly, traditional statistical analysis methods such as logistic regression frequently employed in neonatal risk assessment are based on linear assumptions and are susceptible to challenges like overfitting and multicollinearity. These limitations constrain the ability to examine complex relationships among multiple variables and inhibit the creation of more effective, clinically applicable models (8–10). In contrast, artificial intelligence, particularly machine learning, can overcome these limitations by adapting to large datasets and enhancing predictive performance, allowing for the development of more accurate and flexible clinical models for identifying risk factors in neonatology and other medical fields (11–19).

A C5.0 model is a data mining algorithm that employs a tree-like structure for decision making. It classifies datasets through conditional branching to produce accurate classification results. Decision trees offer efficient data processing capabilities, handling both classification and prediction tasks with highly interpretable and intuitive outcomes (20). Consequently, they provide a reliable approach for comprehensive and nuanced analysis in clinical practice. Decision tree models are widely used in clinical research to develop predictive tools for disease diagnosis and prognosis and to identify key factors influencing disease outcomes (21).

In this study, we retrospectively analyzed data from a national multicenter prospective web-based registry using machine learning algorithms with the C5.0 model to develop a clinical prediction rule for predicting MPH and MPH-related mortality in extremely low birth weight infants (ELBWIs). We compared these findings with those from statistical multivariate regression analysis to appraise the effectiveness of the C5.0 decision tree model in identifying risk factors for MPH and MPH-related mortality.

## 2 Methods

### 2.1 Data source

Data were collected from a de-identified dataset approved by the Committee of Ethics and Publication of the Korean Neonatal Network (KNN). The KNN is a nationwide prospective registration system for VLBW infants admitted to 69 participating hospitals in Korea (22). Data collection for the KNN received approval from the Institutional Review Board (IRB) of each participating hospital, with all data regularly monitored by the KNN data management committee. Written informed consent was obtained from parents upon enrollment at every NICU participating in the KNN.

### 2.2 Study population

We conducted a retrospective cohort study using KNN data from 6,195 preterm infants with BW <1,000 g and GA <32 weeks who were admitted to participating NICUs from January 1, 2013 to December 31, 2020. We excluded 202 infants with any major congenital anomaly and 241 infants with missing data on patent ductus arteriosus (PDA) management. Ultimately, clinical data of 5,752 ELBWIs were included in this study.

### 2.3 Outcome measures

Clinical characteristics of infants included perinatal variables (such as oligohydramnios, multiple gestation, gestational diabetes mellitus (GDM), pregnancy induced hypertension (PIH), premature rupture of membranes (PROM), completion of a full course of antenatal corticosteroids (ACS), and place of birth) and neonatal variables [such as gestational age (GA), birth weight (BW), sex, small for gestational age (SGA), initial resuscitation, Apgar scores (AS) at 1 min and 5 min, clinical risk index for babies (CRIB)-II scores, body temperature at admission, pH and base excess within 1 h after birth, surfactant use, and symptomatic PDA (sPDA)].

MPH was characterized by significant bloody fluid suction through the endotracheal tube associated with acute respiratory failure or cardiovascular collapse. It was presented with fluffy or ground-glass opacities across lung fields on chest radiography (4).

Oligohydramnios was defined by an amniotic fluid index of less than 5. In cases of multiple gestation during the prenatal period with subsequent fetal demise before birth, stillborn babies beyond 16 weeks of gestational age were included in overall counts and classified within the multiple gestation cohort. Conversely, selective abortions of fetuses from *in vitro* fertilization or cases of vanishing twins with gestational age less than 15 weeks and 6 days were excluded from these counts. Pregnancy-induced hypertension (PIH) encompassed diagnoses of gestational hypertension, preeclampsia, or eclampsia. For ACS usage, completion was defined when the full course of steroid treatment, either two doses of betamethasone or four doses of dexamethasone administered 24 h apart, was administered within one week prior to delivery.

SGA was defined as a birth weight below the 10th percentile for GA and sex (23). Initial resuscitation included applying at least one of the following techniques at birth: oxygen supplementation, continuous positive airway pressure (CPAP), positive pressure ventilation, intubation, chest compression, and administration of medications such as epinephrine. Body temperature at admission was defined as the body temperature initially measured in degree Celsius (℃) within an hour of admission. The use of surfactant was designated for prophylactic or rescue treatment of respiratory distress syndrome (RDS) but excluded when it was used for treating pulmonary hypertension or meconium aspiration syndrome. Symptomatic PDA (sPDA) was characterized by the presence of at least three of the following five symptoms associated with PDA, confirmed by a diagnosis of large left-to-right ductal flow by echocardiography: (1) a systolic or continuous murmur; (2) a bounding pulse or hyperactive precordial pulsation; (3) hypotension; (4) respiratory difficulty; and (5) pulmonary edema or cardiomegaly (cardiothoracic ratio >60%) on a chest radiograph (24).

### 2.4 Statistical analysis

During data cleansing, missing data based on MPH criteria were excluded from analysis. Categorical variables were analyzed using the Chi-square (*χ*^2^) test. Variables with frequencies less than five and a prevalence of less than 20% were analyzed using Fisher's exact test. Continuous variables were analyzed using the Mann–Whitney test after checking for normality.

Multivariate regression analysis and decision tree analysis were utilized to examine variables significantly associated with the incidence and mortality of MPH. Variables identified as significant in the univariate analysis (*P* < 0.05) were included in the multivariate analysis. Based on significant variables, a C5.0 decision tree model was developed to improve predictive accuracy.

The dataset was randomly partitioned into two subsets, with 70% allocated for model training and the remaining 30% designated for testing. The model's performance was then evaluated using metrics. The decision tree pruning options included local pruning with a maximum tree depth of 5, a global pruning with a minimum parent node size of 100, and a child node size of 50. These settings were selected to prevent overfitting by restricting tree growth and pruning branches with insufficient data points. Additionally, 10-fold cross-validation was conducted. However, boosting was not applied. These parameters aligned with the SPSS Node Model Options to ensure transparency and reproducibility.

All statistical analyses were conducted using IBM SPSS version 25.0 (IBM Corporation, Armonk, New York, United States). A decision tree was created in IBM Clementine version 10.0 using the C5.0 model. Its performance was evaluated using confusion metrics, including accuracy, precision, and recall.

## 3 Results

**Figure 1:**
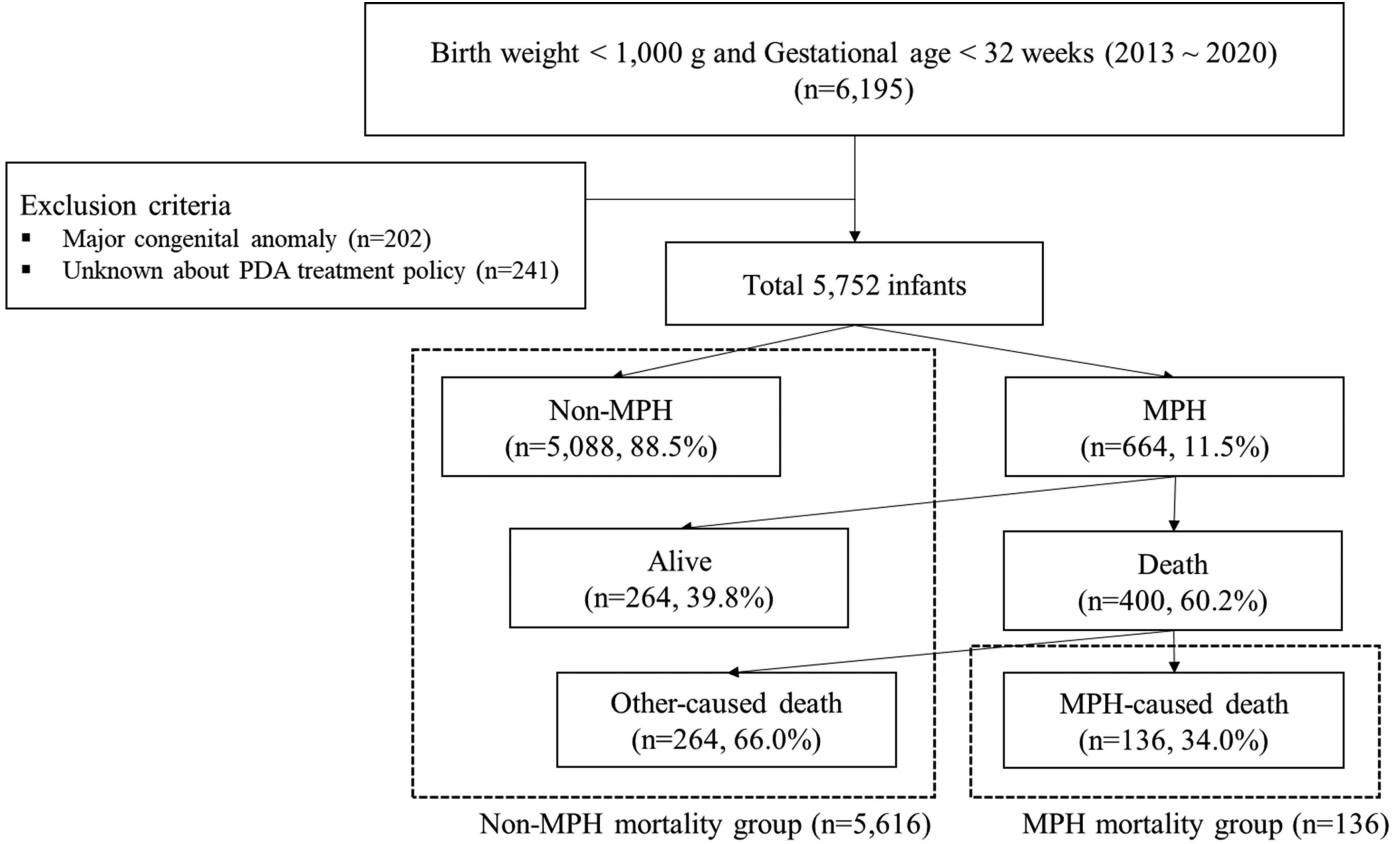
Study population identified with subsequent flow chart of the study in extremely low birth weight infants. PDA, patent ductus arteriosus; MPH, massive pulmonary hemorrhage.

A total of 5,752 ELBWIs were included, with 664 (11.5%) diagnosed with MPH. Infants were categorized into MPH (*n* = 664, 11.5%) and non-MPH groups (*n* = 5,088, 88.5%). Additionally, infants were divided into two categories: those whose deaths were related to MPH (MPH-related mortality group, *n* = 136, 2.4%) and those whose did not (non-MPH mortality group, *n* = 5,616, 97.6%) (Figure 1). In the decision tree model, nodes were split based on the risk factor with the highest Chi-square value and data were recursively partitioned into subgroups to optimize the classification accuracy.

Prenatal and neonatal characteristics and perinatal interventions were compared between MPH and non-MPH groups as well as between the group that died due to MPH (MPH-related mortality group) and the group that survived (non-MPH mortality group) (Table 1).

**Table 1:** Comparison of prenatal and neonatal characteristics and perinatal interventions in MPH, non-MPH, MPH mortality, and non-MPH mortality groups.

Variables	non-MPH (*n* = 5,088, 88.5%)	MPH (*n* = 664, 11.5%)	*P* value	Non-MPH mortality (*n* = 5,616, 97.6%)	MPH mortality (*n* = 136, 2.4%)	*P* value
Oligohydramnios	879 (18.9)	89 (14.9)	.019	958/5,131 (18.7)	10/118 (8.5)	0.005
Multiple gestations	1,646 (32.4)	282 (42.5)	<.001	1,861/5,616 (33.1)	67/136 (49.3)	<0.001
Gestational diabetes mellitus	296 (5.8)	37 (5.6)	.799	328/5,616 (5.8)	5/136 (3.7)	0.286
Pregnancy-induced hypertension	916 (18.0)	119 (17.9)	.959	1,009/5,616 (18.0)	26/136 (19.1)	0.730
Premature rupture of membranes	1,894 (37.5)	222 (33.6)	.052	2,085 5,572 (37.4)	31/135 (23.0)	0.003
Complete course of antenatal steroids	2,494 (49.8)	278 (42.5)	<.001	2,728/5,530 (49.3)	44/133 (33.1)	<0.001
Outborn	154 (3.0)	20 (3.0)	.983	171/5,616 (3.0)	3/136 (2.2)	0.800
Gestational age (weeks)	26^+1^ [24^+6^, 27^+4^]	25^+3^ [24^+3^, 26^+5^]	<.001	26^+0^ [24^+6^, 27^+4^]	25^+1^ [24^+2^, 26^+4^]	<0.001
Birth weight (g)	796 [670, 900]	710 [600, 834]	<.001	790 [660, 900]	680 [570, 790]	<0.001
Sex: male	2,436 (47.9)	363 (54.8)	.001	2,727/5,615 (48.6)	72/136 (52.9)	0.313
Small for gestational age	1,029 (20.2)	152 (22.9)	.109	1,147/5,616 (20.4)	34/136 (25.0)	0.192
Need for initial resuscitation	4,948 (97.8)	657 (99.2)	.014	5,470/5,585 (97.9)	135/136 (99.3)	0.529
APGAR score at 1 min	4.00 [3.0, 5.0]	4.00 [2.0, 5.0]	<.001	4.0 [2.0, 5.0]	3.0 [2.0 5.0]	0.001
APGAR score at 5 min	7.00 [5.0, 8.0]	6.00 [5.0, 7.0]	<.001	7.00 [5.0, 7.0]	6.0 [3.0 7.0]	<0.001
CRIB-II score	11.0 [9.0, 13.0]	11.0 [9.0, 14.0]	<.001	11.0 [9.0, 14.0]	13.0 [11.0 15.0]	<0.001
Body temperature at admission	36.2 [35.8, 36.5]	36.1 [35.7, 36.4]	<.001	36.2 [35.8, 36.4]	36.0 [35.5 36.3]	0.001
pH within 1 h after birth	7.27 [7.19, 7.34]	7.25 [7.18, 7.33]	.037	7.27 [7.19, 7.33]	7.25 [7.18 7.31]	0.065
Base excess within 1 h after birth	−4.6 [−7.0, −2.2]	−5.7 [−8.7, −3.1]	.001	−5.0 [−8.0, −2.8]	−6.00 [−9.0 −4.0]	0.020
Use of surfactant	4,843 (95.2)	658 (99.1)	<.001	5,366/5,616 (95.5)	135/136 (99.3)	0.036
Symptomatic PDA	2,366 (46.5)	394 (59.3)	<.001	2,695/5,616 (48.0)	65/136 (47.8)	0.954

Values are presented as median [range] or number (%).

*P*-values were calculated using the Chi-square (*χ*^2^) tests or the Mann–Whitney test.

MPH, massive pulmonary hemorrhage; CRIB-II score, clinical risk index for babies score; PDA, patent ductus arteriosus.

### 3.1 Factors associated with MPH

Clinical variables in ELBWIs significantly (*P*-value <0.1) associated with MPH were analyzed using multivariate logistic regression. Results are presented in Table 2. Three risk factors associated with an increased incidence of MPH were identified: multiple gestation (HR: 1.595; 95% CI: 1.277–1.992), surfactant use (HR: 4.010; 95% CI: 1.242–12.946), and sPDA (HR: 1.700; 95% CI: 1.357–2.129). In contrast, PROM (HR: 0.709, 95% CI: 0.554–0.909) and higher gestational age (HR: 0.989; 95% CI: 0.980–0.998) were associated with a decreased incidence of MPH.

**Table 2:** Risk factors for MPH and mortality due to MPH in ELBWIs by multivariate logistic regression analysis.

Variables	MPH			MPH mortality		
	Multivariate analysis					
	Exp (B)	95% CI	*P* value	Exp (B)	95% CI	*P* value
Oligohydramnios	0.851	0.632–1.148	0.292	0.424	0.179–1.001	0.050
Multiple gestation	1.621	1.297–2.025	<0.001	1.969	1.228–3.157	0.005
Premature membrane rupture	–	–	–	0.600	0.340–1.058	0.078
Complete course of antenatal steroids	0.775	0.620–0.968	0.024	0.532	0.322–0.879	0.014
Gestational age (weeks)	0.998	0.997–0.999	<0.001	1.001	0.980–1.021	0.956
Sex: male	1.279	1.027–1.592	0.028	–	–	–
Need for initial resuscitation	2.212	0.527–9.282	0.278	–	–	–
APGAR at 1 min	0.993	0.902–1.092	0.878	1.060	0.859–1.307	0.588
APGAR at 5 min	0.926	0.848–1.011	0.085	0.800	0.668–0.959	0.016
Body temperature at admission	1.027	0.882–1.197	0.730	1.068	0.783–1.455	0.679
pH within 1 h after birth	2.002	0.612–6.550	0.251	–	–	–
Base excess within 1 h after birth	0.976	0.944–1.008	0.142	1.003	0.951–1.057	0.916
Use of surfactant	4.695	1.450–15.199	0.010	18,363,410.67	0.000-	0.995
Symptomatic PDA	1.772	1.375–2.157	<0.001	–	–	–

Adjusted for variables (except BW) with *P*-values less than 0.05.

“Use of surfactant” was excluded from the multivariable analysis due to its extremely low frequency in MPH mortality cases (1/136), which resulted in unstable exp(B) estimates.

MPH, massive pulmonary hemorrhage; PDA, patent ductus arteriosus.

### 3.2 Factors associated with mortality-related MPH

Clinical variables in ELBWIs associated with MPH-related mortality having *P*-values <0.1 were analyzed using multivariable analysis. Results are presented in Table 2. Multiple gestation (HR: 1.878; 95% CI: 1.172–3.008) was found to be a significant risk factor for increased mortality due to MPH. Conversely, PROM (HR: 0.540; 95% CI: 0.308–0.949), a complete course of ACS (HR: 0.566, 95% CI: 0.343–0.935), and a higher APGAR score at 5 min (HR: 0.800; 95% CI: 0.667–0.960) were associated with decreased mortality for patients with MPH.

### 3.3 C5.0 decision prediction of perinatal factors associated with MPH

Our multivariate logistic regression analysis identified lower GA, multiple gestation, surfactant use, and symptomatic PDA as independent risk factors for MPH in a total of 5,752 ELBWI. Our results aligned with results of our previous study (1) on VLBWIs except that small for gestational age was not identified in the current study as a risk factor for MPH.

Multivariate analysis was performed to determine independent effects of various variables on MPH. Results confirmed the relative importance of specific factors, including gestational age (GA), birth weight, and APGAR scores. Upon examining potential effects of multicollinearity, we found that gestational age (GA) had a Variance inflation factor (VIF) of 8.3, birth weight had a VIF of 6.7, APGAR score at 1 min had a VIF of 5.2, and APGAR score at 5 min had a VIF of 5.8. Although multicollinearity was present, it was determined that model adjustment was unnecessary, as all values were below the threshold of 10.

Regarding the predicted accuracy of the C5.0 MPH model, it exhibited an AUC of 0.882 on the training set and an AUC of 0.890 on the validation set.

**Figure 2:**
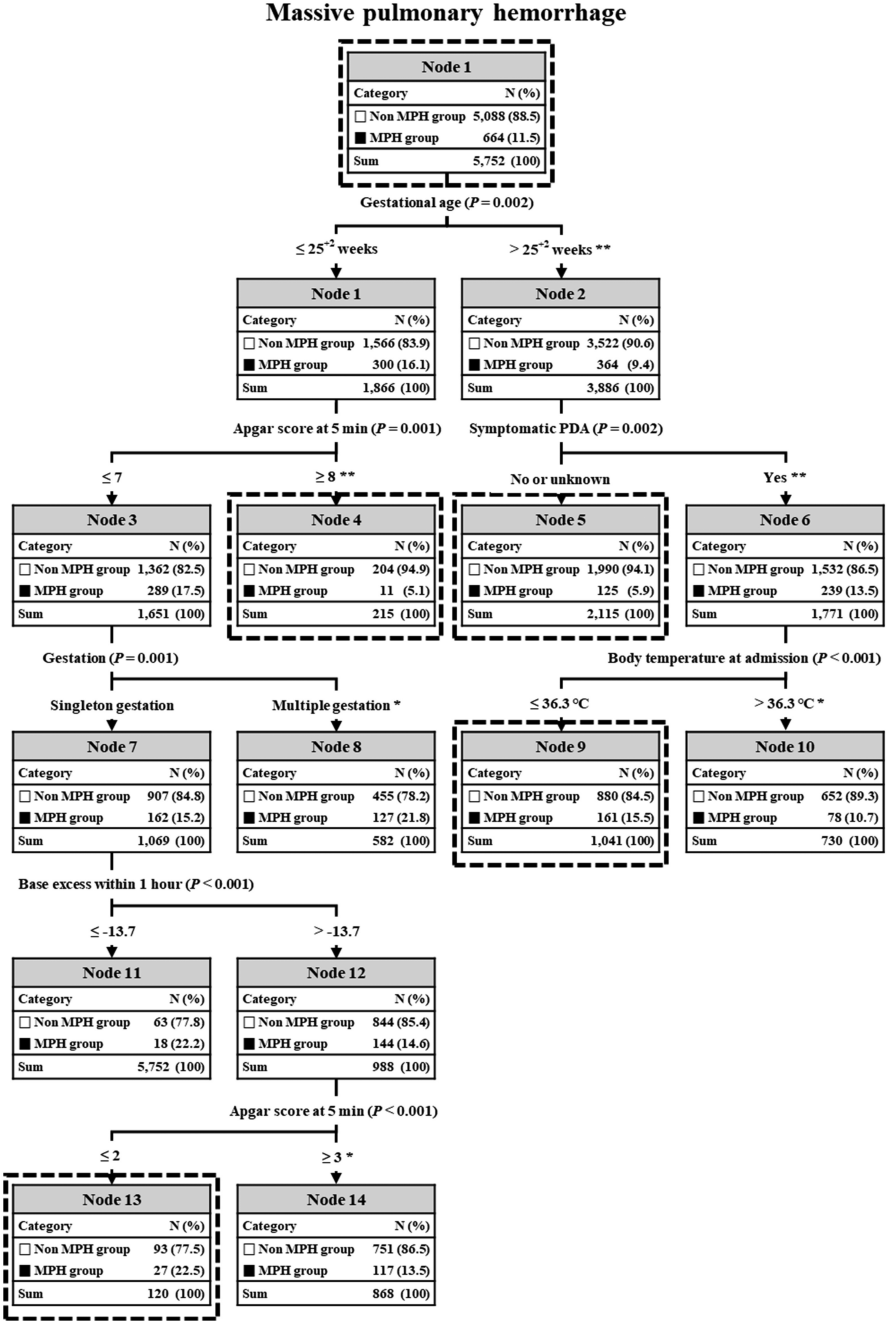
C5.0 decision prediction of all factors in MPH. In C5.0, the *P*-value from a chi-square test or Gini index indicates a variable’s significance in decision splitting. The asterisk* represents a *P*-value from the chi-square test for each node's frequency. *P value < 0.01, **P value < 0.001. MPH, massive pulmonary hemorrhage; PDA, patent ductus arteriosus.

The C5.0 decision prediction of perinatal factors influencing MPH was demonstrated in Figure 2. It included six risk factors: GA, APGAR score at 5 min, symptomatic PDA, type of pregnancy, body temperature at admission, and base excess within 1 h after birth. The incidence of MPH significantly differed at each node categorized by these risk factors, as evidenced by the chi-square test. Infant population was divided into 8 subgroups through different branches from the root node to the leaf node in the C5.0 analysis.

GA was the primary factor distinguishing a higher incidence of MPH among the six factors. In preterm infants with “gestational age ≤25^+2^”, the next discriminator was “APGAR score at 5 min  ≤ 7.5”, followed by “multiple gestation”. In case of “single gestation”, the next discriminator was “base excess within 1 h after birth ≤−13.7”, with the final discriminator being “APGAR score at 5 min ≤2.5”. Consequently, node 15 in “single gestation” and node 8 in “multiple gestation” displayed the highest incidences of MPH at 22.5% and 21.8%, respectively, while node 4 exhibited the lowest incidence of MPH at 5.1%.

On the other hand, for preterm infants with “gestational age ‘>25^+2^”, the next discriminator was “Symptomatic PDA” and the terminal discriminator was “BT at admission ≤36.0”. Consequently, node 9 displayed the highest incidence of MPH at 15.5% and node 5 showed the lowest incidence of MPH at 5.9%. A chi-square test evaluating predicted frequencies of decision tree nodes showed statistical significance in all nodes except for MPH nodes 11 and 12.

### 3.4 C5.0 decision prediction of perinatal factors associated with MPH-related mortality

**Figure 3:**
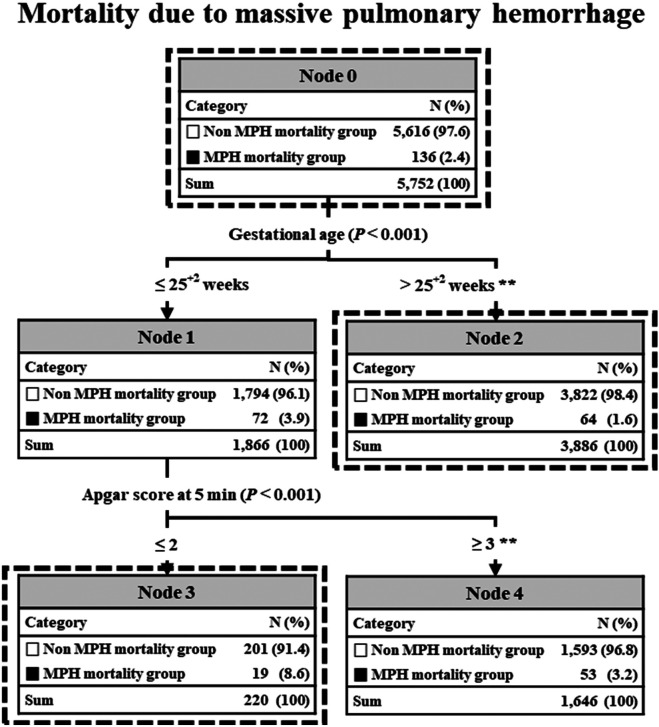
C5.0 decision prediction of all factors in MPH-related mortality. In C5.0, the *P*-value from a chi-square test or Gini index indicates a variable's significance in decision splitting. The asterisk* represents a *P*-value from the chi-square test for each node's frequency. *P value <0.01, **P value <0.001. MPH, massive pulmonary hemorrhage.

Regarding the predicted accuracy of the C5.0 MPH-related mortality model, it demonstrated an AUC of 97.7% on the training set and an AUC of 97.4% on a validation set. Results of C5.0 decision prediction of neonatal factors for MPH-related mortality are depicted in Figure 3. Two risk factors, GA and APGAR score at 5 min, were included. The incidence of MPH at each node categorized by each risk factor showed significant differences according to the chi-square test. Infant populations were divided into three subgroups using different branches from the root node to the leaf node in the C5.0 analysis. GA was the primary discriminator for the high incidence of MPH-related mortality among these factors.

In preterm infants with “gestational age ≤25^+2^”, the next discriminator was “APGAR score at 5 min ≤2.5”. Consequently, node 3 exhibited the highest incidence of MPH-related mortality at 8.6%, while node 4 had the lowest incidence at 3.2%. On the other hand, in preterm infants with “gestational age ‘>25^+2^”, there were no subsequent discriminators. Consequently, node 2 exhibited the lowest incidence of MPH-related mortality at 1.6%. After conducting a chi-square test to evaluate predicted frequencies of decision tree nodes, all nodes were found to be statistically significant.

## 4 Discussion

To the best of our knowledge, this is the first study that employs a decision tree analysis using the C5.0 algorithm to develop a clinical prediction model for MPH and MPH-related mortality, utilizing a population-based cohort study design with a large number of ELBWIs in NICUs. Besides identifying risk factors, this approach also established a cut-off value for each, effectively classifying subgroups with statistically significant differences in incidences of MPH and MPH-related mortality.

### 4.1 Risk factors for MPH

In our decision tree analysis utilizing the C5.0 model, six risk factors were identified for MPH, including GA, APGAR score at 5 min, symptomatic PDA, multiple gestation, body temperature at admission, and base excess within the first hour after birth.

Decreasing gestational age and birth weight are consistently cited in the literature as clear risk factors for mortality and morbidity in extremely preterm infants (25, 26). They are also recognized as major risk factors for MPH and MPH-related mortality in ELBWIs (5, 27). In our previous studies, MPH and MPH-related mortality were observed in 870 (6.3%) and 162 (1.2%) of VLBWIs and in 664 (11.5%) and 136 (2.4%) of ELBWIs registered in the Korean Neonatal Network (KNN) from 2013 to 2020, respectively (3).

Previous studies identifying risk factors for MPH primarily used statistical methods such as logistic regression. In most studies (3, 5, 6), gestational age and birth weight were significant factors in univariate analysis but not independent predictors in multivariate analysis. This suggests that these factors might be confounded by other variables or lack a direct effect on the outcome when controlling for other variables. Therefore, despite their clinical importance, the significance of gestational age and birth weight might have been underestimated and misrepresented by current statistical methodologies.

However, our decision tree analysis revealed that GA was the primary discriminator for predicting the occurrence of MPH among six risk factors. Interestingly, this analysis determined a GA cut-off of “25 weeks and 2 days” for a high incidence of MPH. As a result, the incidence of MPH in Node 1 (GA ≤ “25 weeks and 2 days”) and Node 2 (GA > “25 weeks and 2 days”) showed a statistically significant difference, as indicated by the chi-square test (16.1% vs. 9.4%, *P* < 0.001).

#### 4.1.1 Preterm infants with a gestational age ≤25 weeks and 2 days

According to our decision tree analysis, preterm infants with a gestational age ≤25 weeks and 2 days were then assessed based on their APGAR score at 5 min, with a cut-off value of 7.5, indicating a high incidence of MPH. Consequently, statistically significant differences were observed in the incidence of MPH in Node 3 (APGAR score at 5 min ≤7) and Node 4 (APGAR score at 5 min ≥8), as demonstrated by the chi-square test (17.5% vs. 5.1%, *P* < 0.001). Although the APGAR score has been identified as a statistically significant factor in univariate analysis across multiple studies (3, 5, 7), including ours, it has not been confirmed in multivariate analysis. Therefore, despite its clinical relevance, the importance of the APGAR score might have been underestimated and misrepresented by current statistical methods.

For preterm infants with a gestational age ≤25 weeks and 2 days and an APGAR score at 5 min ≤7, the type of pregnancy emerged as the next key discriminator. There was a significant difference in MPH incidence between single gestations (Node 7) and multiple gestations (Node 8), as indicated by the chi-square test (15.2% vs. 21.8%, *P* < 0.01). Multiple gestations associated with higher risks and complications compared to single gestations have been reported to significantly increase the incidence of MPH and related mortality (4, 28).

Although few studies have considered multiple pregnancies as a perinatal factor, both past and recent research (3) has confirmed its statistical significance in both univariate and multivariate analyses.

In our decision tree analysis, Node 8 (multiple gestation) was identified as a leaf (terminal) node, showing no further subdivisions despite a high incidence of MPH and a substantial number of affected infants. This underscores the clinical importance of multiple gestation.

Node 7 (single gestation) is an internal node that splits the data based on “base excess within 1 h after birth” with a cut-off value of −13.7 indicating a high incidence of MPH. Consequently, a statistically significant difference in the incidence of MPH was observed between Node 11 (base excess ≤−13.7) and Node 4 (base excess >−13.7), as shown by the chi-square test (22.2% vs. 14.6%, *P* < 0.001). Previous research (3) has found that base excess score is a statistically significant factor in univariate analysis, although it is not a significant factor in multivariate analysis. Therefore, despite its clinical relevance, the significance of base excess score might have been underestimated and misrepresented by current statistical methodologies.

The incidence of MPH is higher in infants born at less than 25 weeks gestation, particularly in those with low 5-minute Apgar scores and low base excess (BE). Therefore, proactive management including active implementation of neonatal resuscitation (NRP) is essential for improving 5 min Apgar scores and BE in these high-risk infants.

#### 4.1.2 Preterm infants with a gestational age >25 weeks and 2 days

According to our decision tree analysis, in preterm infants with a gestational age >25 weeks and 2 days, the next discriminator was the presence of symptomatic PDA for the high incidence of MPH. Consequently, the incidence of MPH in Node 6 (with symptomatic PDA) and Node 5 (without symptomatic PDA) showed a statistically significant difference, as demonstrated by the chi-square test (13.5% vs. 5.9%, *P* < 0.001). Almost all studies (3, 7), including ours, identified the presence of symptomatic PDA as a statistically significant factor in both univariate and multivariate analyses. Therefore, proactive management to decrease the risk of sPDA should be recommended as preventive strategies to reduce the risk of MPH in preterm infants with a gestational age >25 weeks and 2 days.

Node 6 (with symptomatic PDA) is an internal node that splits the data based on “body temperature at admission” with a cutoff value of 36°C for a high incidence of MPH. As a result, the incidence of MPH in Node 9 (body temperature ≤36°C) and Node 10 (body temperature >36°C) showed a statistically significant difference as demonstrated by the chi-square test (15.5% vs. 10.7%, *P* < 0.05). In our previous study (3), body temperature was a statistically significant factor in univariate analysis but not in multivariate analysis. However, despite its clinical importance, the significance of body temperature might be underestimated and misrepresented by current statistical methodologies.

In infants born at 25 weeks or later, the incidence of MPH increased in the presence of sPDA and low body temperature. Therefore, proactive management for decreasing the risk of sPDA at the beginning of life and maintaining optimal body temperature should be recommended as preventive strategies for reducing the risk of MPH in extremely preterm infants.

### 4.2 Risk factors for MPH-related mortality

In our multivariate logistic regression analysis, multiple gestation and APGAR score at 5 min were identified as ELBWIs independent risk factors for MPH-related mortality. These findings are consistent with those of our previous study (3) on VLBWIs in the same cohort database.

In our decision tree analysis using the C5.0 model, GA and APGAR score at 5 min were identified as two risk factors. Our decision tree analysis identified GA as the primary discriminator for predicting the occurrence of MPH-related mortality. Intriguingly, this analysis provided a cutoff value of GA at “25 weeks and 2 days” for a high incidence of MPH-related mortality. Consequently, the incidence of MPH-related mortality in Node 1 (GA ≤ “25 weeks and 2 days”) and Node 2 (GA > “25 weeks and 2 days”) demonstrated a statistically significant difference, as evidenced by the chi-square test (3.9% vs. 1.6%, *P* < 0.001).

In preterm infants with a gestational age ≤25 weeks and 2 days, the APGAR score at 5 min served as the next discriminator, with a cut-off value of 2.5 indicating a high incidence of MPH-related mortality. Consequently, the incidence of MPH-related mortality in Node 3 (APGAR score at 5 min ≤2) and Node 4 (APGAR score ≥3) exhibited a statistically significant difference, as evidenced by the chi-square test (8.6% vs. 3.2%, *P* < 0.001). APGAR scores were statistically significant factors in both univariate and multivariate analyses in almost all studies (3, 4, 7), including our study.

In preterm infants with a gestational age >25 weeks and 2 days, Node 2 was identified as a terminal node that could not split further due to the lowest incidence of MPH-related mortality. This finding indicates that GA plays a clinically significant role in MPH-related mortality, suggesting that a higher gestational age might be associated with a decreased risk of mortality associated with MPH.

MPH-related mortality was also found to be significantly higher in infants born at less than 25 weeks of gestation with low 5 min. Apgar scores. This suggests that proactive NRP focused on improving 5 min. Apgar scores might contribute to reduced mortality associated with MPH.

### 4.3 Clinical implications of the decision tree analysis

Traditional statistical analysis methods such as logistic regression have been widely used in neonatal risk assessment due to their interpretability and ease of implementation. However, these analyses rely on linear assumptions, which may limit their ability to capture complex, non-linear relationships between variables. Additionally, they are susceptible to challenges such as overfitting and multicollinearity, potentially compromising the accuracy and generalizability of findings. These inherent limitations restrict the ability to analyze intricate interactions among multiple factors, which are often critical in clinical decision-making.

In contrast, decision tree models as a decision-making tool can provide a more flexible and robust alternative by addressing these challenges. Unlike traditional statistical analysis, decision trees do not require strict linearity assumptions. They can accommodate complex, non-linear relationships within the data. Furthermore, they offer enhanced interpretability by visually representing variable interactions, which can facilitate the identification of key predictors and their relative importance in clinical outcomes.

Given these advantages, decision tree models may provide more practical insights for neonatal risk assessment, ultimately enhancing predictive accuracy and supporting more informed clinical decision-making. Future studies should explore integration of advanced machine learning approaches, such as ensemble methods and deep learning, to further refine predictive capabilities and improve neonatal care outcomes.

### 4.4 Limitations

Our study has several limitations. Firstly, the retrospective analysis of a large population-based cohort resulted in a small number of deaths which restricted our ability to identify statistically significant mortality risk factors, although the overall sample size of our study was substantial. Secondly, the presence of null values in the KNN data presumably influenced the performance of the C5.0 model, thereby diminishing its safety and reliability. Thirdly, biological interactions between perinatal and neonatal factors are complex. Many uncontrollable factors and individual biological variability can affect patient prognosis. Therefore, coupling this algorithm with complementary methods, performing rigorous data preprocessing, and conducting ongoing validation to ensure reliable and actionable predictions in clinical settings are crucial. Especially, a potential limitation of this study is the presence of multicollinearity among key predictor variables such as gestational age, birth weight, and APGAR scores. Given their known correlations, multicollinearity might have influenced the loss of statistical significance for certain variables in the multivariable logistic regression model. In addition, we did not explicitly calculate Variance Inflation Factors (VIF). This limitation should be considered when interpreting our results. Future studies may benefit from additional statistical techniques such as collinearity diagnostics. In addition, our study utilized retrospectively collected data from a population-based cohort study. Moreover, our model was not externally validated. Consequently, future research should aim to externally validate the model using prospective data from diverse populations to improve its generalizability and reliability. Additionally, further investigations should focus on integrating real-time data and employing more sophisticated algorithms to enhance predictive accuracy of the C5.0 model.

## 5 Conclusions

In this study, we developed a decision tree model based on clinical risk factors to predict MPH and MPH-related mortality in ELBWIs. This predictive approach enabled early identification of infants at high risk of MPH and MPH-related mortality, facilitating timely and targeted interventions. By optimizing treatment strategies and enhancing clinical management, it ultimately improved neonatal outcomes.

Decision-based risk stratification tools using a decision tree model could be incorporated into neonatal intensive care protocols. However, further validation with larger, multicenter cohorts is essential for evaluating the practical applicability and clinical effectiveness of the C5.0 decision tree model in NICUs before routine clinical implementation.

## Data Availability

The original contributions presented in the study are included in the article/Supplementary Material, further inquiries can be directed to the corresponding author.
